# Biomarkers of Castrate Resistance in Prostate Cancer: Androgen Receptor Amplification and T877A Mutation Detection by Multiplex Droplet Digital PCR

**DOI:** 10.3390/jcm11010257

**Published:** 2022-01-04

**Authors:** Francis P. Young, Therese M. Becker, Mohammed Nimir, Thomas Opperman, Wei Chua, Bavanthi Balakrishnar, Paul de Souza, Yafeng Ma

**Affiliations:** 1 Centre for Circulating Tumour Cell Diagnostics and Research, Ingham Institute for Applied Medical Research, Liverpool, NSW 2170, Australia; francis.p.young@icloud.com (F.P.Y.); t.becker@unsw.edu.au (T.M.B.); mohammed.a.nimir@gmail.com (M.N.); t.opperman@student.unsw.edu.au (T.O.); 2 South Western Clinical School, University of New South Wales, Liverpool, NSW 2170, Australia; p.desouza@westernsydney.edu.au; 3 School of Medicine, Western Sydney University, Campbelltown, NSW 2560, Australia; Wei.Chua@health.nsw.gov.au; 4 Liverpool Hospital, Liverpool, NSW 2170, Australia; Bavanthi.Balakrishnar@health.nsw.gov.au

**Keywords:** prostate cancer, cell free DNA, liquid biopsy, androgen receptor, mutation, amplification

## Abstract

Androgen Receptor (AR) alterations (amplification, point mutations, and splice variants) are master players in metastatic castration resistant prostate cancer (CRPC) progression and central therapeutic targets for patient management. Here, we have developed two multiplexed droplet digital PCR (ddPCR) assays to detect AR copy number (CN) and the key point mutation T877A. Overcoming challenges of determining gene amplification from liquid biopsies, these assays cross-validate each other to produce reliable AR amplification and mutation data from plasma cell free DNA (cfDNA) of advanced prostate cancer (PC) patients. Analyzing a mixed PC patient cohort consisting of CRPC and hormone sensitive prostate cancer (HSPC) patients showed that 19% (9/47) patients had AR CN amplification. As expected, only CRPC patients were positive for AR amplification, while interestingly the T877A mutation was identified in two patients still considered HSPC at the time. The ddPCR based analysis of AR alterations in cfDNA is highly economic, feasible, and informative to provide biomarker detection that may help to decide on the best follow-up therapy for CRPC patients.

## 1. Introduction

Prostate cancer (PC) remains one of the leading causes of cancer related deaths in the western world. Molecular profiles indicate that increased androgen receptor (AR) activity is one of the main drivers during PC development. Therefore, PC responds generally well to androgen deprivation therapy (ADT). However, eventually PC develops ADT resistance mechanisms, commonly via AR mutations, amplification, and splice variants [1]. There is an active area of developing next generation ADTs with efficacy even in the context of these AR alterations, which warrants fast and practical identification of these alterations and will have the potential to underpin clinical trials to define patient eligibility and outcomes.

AR amplification correlates with castration resistant prostate cancer (CRPC) and is found in approximately 20–60% of PC recurring during ADT while very rare in primary PC (<1%) [2]. Variation in detection frequency is attributed to varying patient cohorts and detection techniques with varying sensitivity and specificity using various biopsies [3,4,5,6,7]. AR amplification has commonly been associated with resistance developing against the third line ADT drug enzalutamide, which is a current therapy option for advanced PC patients [8,9].

AR mutations, especially if affecting exon 8 of the AR gene and the ligand binding domain (LBD) of the AR protein, can modulate ligand and cofactor affinity, and result in gain-of-function through increased sensitivity to other steroid ligands (e.g., progesterone, adrenal androgens) [10] or the conversion of direct inhibitors (e.g., bicalutamide and hydroxyflutamide) into agonists [11,12]. However, mutations affecting the AR LBD were found in only 10–20% CRPC patients [8]. The most common and well-studied mutation, AR T877A (sometimes referred to as T878A dependent on codon count consistency), affects both ligand affinity and ligand dose dependency for peptide recruitment [12,13].

It is generally difficult to obtain advanced PC tissue biopsies for biomarker evaluation, in part due to anatomical locations of metastases such as in the bone. Liquid biopsy is emerging as a surrogate non-invasive and economic resource for biomarker detection with high potential, as repeated liquid biopsies are highly feasible and allow longitudinal monitoring of biomarker changes during disease progression. There is also evidence that liquid biopsy may overcome the bias of inter- and intra- tumor heterogeneity and sample accessibility, especially in the advanced PC setting [14]. In addition, current data suggest that common PC biomarkers may be diagnostically screened for from urine or blood [15]. For instance, commonly diagnostically tested prostate specific antigen (PSA) and prostate cancer antigen 3 (PCA3) may be complemented by the analysis of circulating tumor cells (CTCs), circulating tumor nucleic acids (ctDNA and ctRNA), or exosomes for molecular biomarkers, such as the AR-variant 7 (AR-V7) [16,17,18,19]. CTC AR-V7 has been proposed to link to ADT resistance but does not affect response to taxane therapy [18,20,21]. Furthermore, CTC enumeration and plasma AR amplification or AR CN status may be prognostic and associated with poor survival, while changes in CTC number indicate response to therapy or relapse [22].

Here, we developed multiplexed ddPCR assays to analyze AR copy number (CN) and the most common AR T877A mutation using cell free DNA (cfDNA) isolated from plasma of advanced PC patients and detected both markers in our proof of concept patient cohort.

## 2. Materials and Methods

### 2.1. Cell Lines and Healthy Donor PBMC

Prostate cancer cell line LNCaP (T887A+, no AR amplification), VCaP (high AR CN) [23], and mammary carcinoma cell line MFM-223 (medium AR CN) [24] were maintained as previously described [25]. Cell lines were authenticated (AGRF, Melbourne, Australia) and tested to be mycoplasma negative using MycoAlert mycoplasma detection kit (Lonza, Basel, Switzerland). Healthy blood donors were recruited with written consent and healthy PBMCs were obtained after red blood cells removal with Lymphoprep^TM^ (Stem Cell Technologies, Tullamarine, Australia) gradient centrifugation as described before [25].

### 2.2. Plasma

PC patients were recruited from Sydney St. George and Liverpool Hospitals. Patients and healthy controls gave written consent under the ethic HREC/13/LPOOL/158 approved via the South Western Sydney Local Health District Ethics Committee. Peripheral blood was drawn into 9 mL BD Vacutainer EDTA tubes (Becton, Dickinson and Company, Franklin Lakes, NJ, USA) and processed with a double spin protocol as described before [25]. Briefly, within 4 h post draw, the whole blood was spun at 280× *g*, 10 min at room temperature within 4 h and supernatants were transferred and spun at 16,000× *g*, 10 min at 4 °C. Plasma were aliquoted and stored at −80 °C till further use.

### 2.3. Genomic DNA and Plasma cfDNA Extraction

Genomic DNA (gDNA) from PBMC and cell lines were extracted with Bioline genomic DNA extraction kit according to manufacturer’s instructions (Meridian Bioscience, Eveleigh, Australia). gDNA concentration and quality were determined with NanoDrop 2000 (Thermo Fisher Scientific, Waltham, MA, USA). Plasma cfDNA extraction was performed with QIAamp circulating nucleic acid kit plus QIAvac 24 Plus vacuum manifold (Qiagen, Hilden, Germany) as described previously [19,26].

### 2.4. Droplet Digital PCR

Droplet digital PCR (ddPCR) was performed with a Bio-Rad QX200 droplet digital PCR system (droplet generator and C1000 Touch thermocycler and droplet reader). All assays were optimized firstly by determining the best annealing temperature using a temperature gradient (52–60 °C) revealing 57 °C as the best annealing temperature for multiplexed reactions. Primers and probes for AR and appropriate reference genes (Table 1) were designed with Primer 3 and Nucleotide BLAST and ordered from Integrated DNA Technologies (Singapore). Primer and probe concentrations were 500 nM and 250 nM, respectively, unless stated differently (see Table 1), as optimal primer and concentration were determined with probe concentration range (62.5–500 nM) for multiplexing to improve cluster separations. PCR reactions were amplified at 95 °C for 10 min, 94 °C for 30 s, and 57 °C for 1 min for 40 cycles at a ramp rate of 2 °C/s and deactivated at 98 °C for 10 min. For ddPCR template input, about 5 ng cell line or PBMC gDNA were used, while, for extracted cfDNA, 7 µL (for AR-Amp-1/T877A assay) or 3 µL (for AR-Amp-2 assay) were used. After PCR amplification and droplet read, the data were analyzed with QuantaSoft V1.7.4 (Bio-Rad Laboratories, Australia). The thresholds were set and adjusted according to the positive and negative controls in each run. Positive controls are LNCaP cell gDNA (“normal” AR CN, carries T877A mutation) and negative controls include water and T877A negative cell lines (VCaP and MFM-233) and plasma cfDNA from healthy donors. The relative concentrations were shown as copy/µL reaction. Copy number (CN) was calculated as follows:

AR-Amp-1/T877A assay: AR CN = (WT + MT)/RPP300 × 2

AR-Amp-2: AR CN = (AR-X1 + AR-X2)/(MYM* + TBP) × 2

where MYM* is double the MYM raw reading for male samples due to the X-chromosomal hemizygosity for males.

### 2.5. Statistics

Data and figures were analyzed and generated using GraphPad Prism 8 (GraphPad Software, Inc., San Diego, CA, USA). Correlation analysis of 2 methods were performed with Pearson r and different groups (amplification positive vs. healthy control or amplification negative) were compared with a Mann–Whitney test.

## 3. Results

### 3.1. T877A Mutation and AR Amplification Assay (AR-Amp-1/T877A)

**Figure 1:**
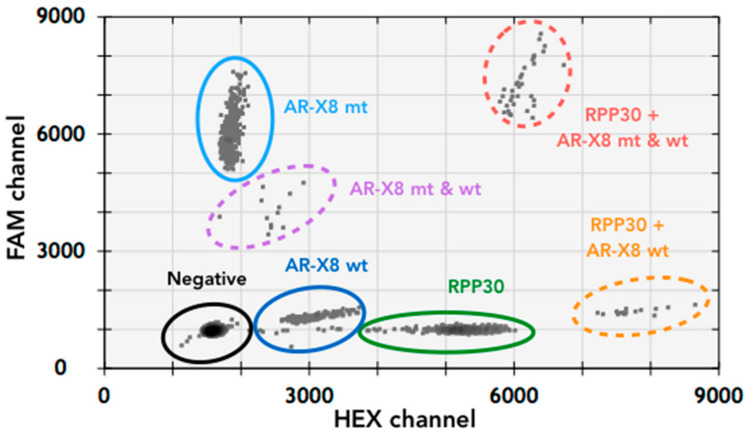
Two-dimensional plot of multiplexed ddPCR AR-Amp-1/T877A assay to detect both the point mutation T877A as well as AR CN. A combination of LNCaP and MFM-233 gDNA was used to represent the various resulting droplet populations. The droplet populations indicated by various colors demonstrate good separation of different variants. Note that the appearance of combination populations is dependent on the amount of DNA input and chance of co-localization of templates within the same droplets.

The AR-Amp-1/T877A assay multiplexes amplification and detection of the AR sequences encoding the T877A mutation by using a T877A specific probe (FAM conjugated) and the corresponding wild-type probe (HEX conjugated). Another set of primers and a probe (HEX conjugated) to detect the reference gene RPP30 located on human chromosome 10 is included to normalize for AR amplification status. For assay optimization, gDNA from cell lines, LNCaP (AR point mutation T877A+) as well as from VCaP and MFM-233 cells (AR amplification positive), were used. Annealing temperature 57 °C with “standard” 500 nM primers and 250 nM probes resulted in the best separation of the four main event populations: negative (empty droplets, without template), T877A (MT), wild-type (WT), and RPP30 (Figure 1). The identity of each event population was confirmed by running assays with individual sets of probes and primers (data not shown). Additional populations (WT + RPP30, WT + MT and RPP30 + WT + MT, see Figure 1) may also be clearly identified when multiplexing and different templates coincide in the same droplet. AR-Amp-1/T877A demonstrated male and female PBMCs had one and two AR CN, respectively (Table 2), due to X-chromosomal localization of AR [2]. Both VCaP cells and MFM-223 are known to carry increased AR CN [23,27] and indeed AR-Amp-1/T877A shows AR CN of 28 and 8, respectively. Furthermore, AR-Amp-1/T877A confirmed that LNCaP cells carry a T877A mutation and no AR CN increase. The assay was able to accurately identify AR CN and the T877A mutation in gDNA from cell lines, even when LNCaP gDNA was spiked into male PBMC, MFM-223, and VCaP gDNA in a 2:1 ratio (Table 2). Interestingly, RPP30 concentration in LNCaP (228 copy/µL) is highly elevated compared to other cells (51.5–90 copy/µL), which reflects its reported polyploid state [28] (Table 2).

### 3.2. AR Amplification by AR-Amp-2 Assay

Given the challenge to reliably detect AR amplification status with high sensitivity and specificity from plasma derived cfDNA, we decided to improve assay accuracy by developing a second multiplexed validation assay (AR-Amp-2) based on DNA amplification with two sets of AR specific primers and probes (both FAM conjugated) targeting two independent AR amplicons: AR exon 1 (AR-X1) and exon 2 (AR-X2). The three AR-loci used in our two assays are distinct and separated from each other by approximately 75,000 base pairs, to ensure coverage of the AR gene. AR-Amp-2 includes primers and probes (both HEX conjugated) independently targeting two reference genes: X-chromosomal zinc finger MYM-type protein 3 (MYM) and TATA-binding protein (TBP) located on chromosome 6.

**Figure 2:**
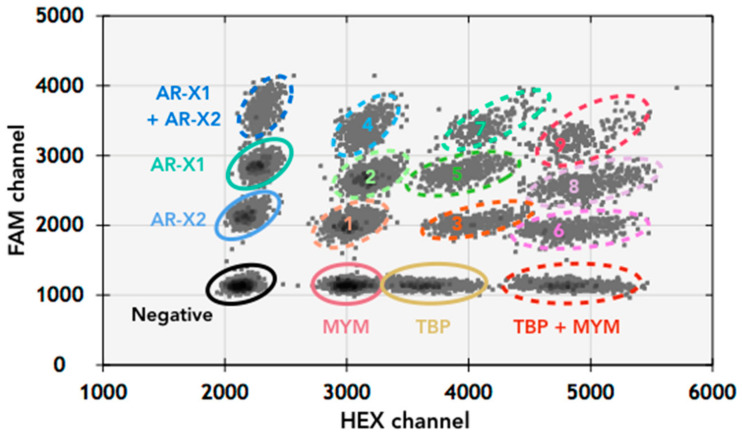
Two-dimensional plot of multiplexed AR-Amp-2 ddPCR assay. The individual droplet clusters demonstrated good separation of different amplicons as shown in the representative 2D plot. Combination populations are also present: 1: AR-X2 + MYM; 2: AR-X1 + MYM; 3: TBP + AR-X2; 4: AR-X1 + X2 + MYM; 5: AR-X1 + TBP; 6: AR-X2 + TBP + MYM; 7: AR-X1 + X2 + TBP; 8: AR-X1 + TBP + MYM; 9: AR-X1 + X2 + TBP + MYM. Note that the appearance of combination populations is dependent on DNA input and chance of co-localization of templates within the same droplets.

To develop this assay, individual probe concentrations were first titrated to identify the corresponding fluorescent amplitude. Increasing population density and separation between fluorescent and background events was expectedly observed with higher probe and primer concentrations (Figure S1). Primers and probes were then multiplexed together with the established concentrations to verify a clean separation of well-defined populations, where four distinct populations of droplets with single templates were distinguishable in each channel (Figure 2), indicating the different combinations of target genes. We were also able to clearly call populations of droplets that had mixed templates (Figure 2). With the AR-Amp-2 assay, AR CN of PBMC and LNCaP were confirmed to be identical to AR-Amp-1/T877A, while AR-Amp2 produced slightly lower AR CN, of 23 (AR-Amp-1: 28) and 7 (AR-Amp-1: 8) for VCaP and MFM-233, respectively (Table 3).

### 3.3. AR Alterations Detected from Patient Plasma

**Figure 3:**
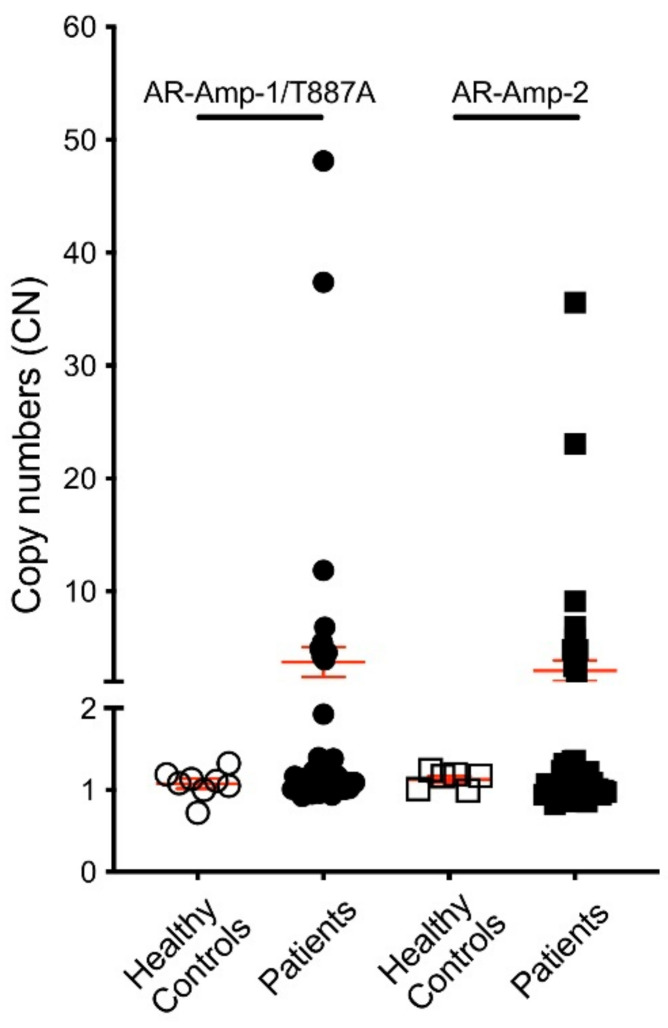
AR CN of 6–8 healthy controls and 47 PC patients. CN was calculated as described in the Materials and Methods section. Circles: AR-Amp-1/T877A; squares: AR-Amp-2; open circles/squares: healthy blood donors; solid circles/squares: patients; error bars in red: mean *±* SEM.

**Figure 4:**
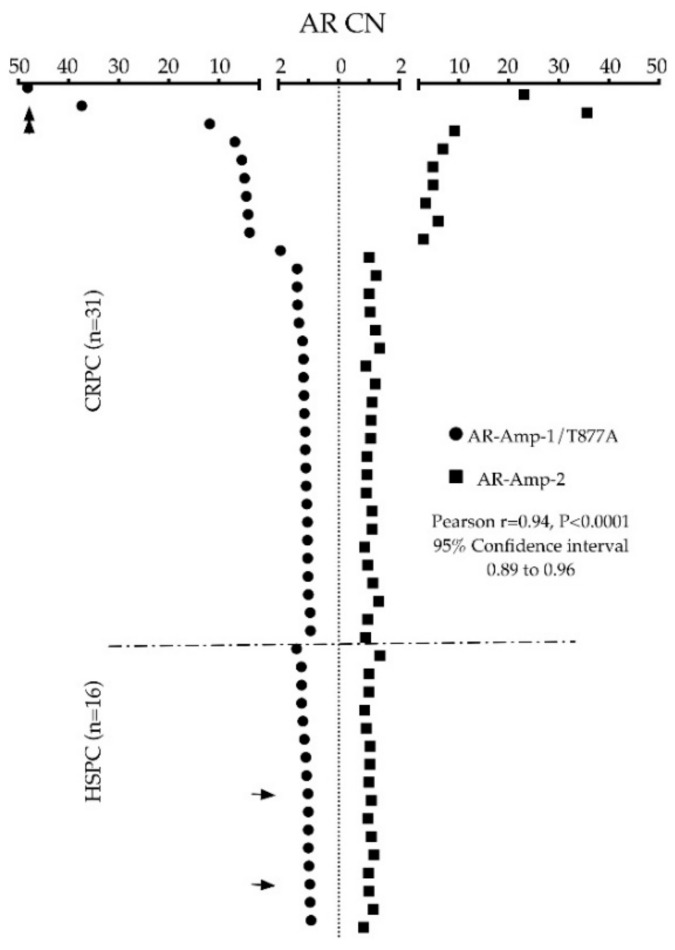
Side-by-side scatter dot plot demonstrating cross-validation of AR CN between AR-Amp-1/T877A (solid circles) and AR-Amp-2 (solid squares) separated into HSPC vs. CRPC patient groups. Patient sample data are sorted by CN value measured by the AR-Amp-1/T877A method. Arrows indicate patient samples with identified T877A mutation, double arrows indicate patients with large scale Chromosome X CN increase.

To validate translation of our assays, we first tested cfDNA derived from 6–8 healthy donor plasma for threshold setting before applying our assays in a 47 PC patient cohort. Clinicopathological characteristics of patients are listed in Supplementary Table S1. Of note, the cohort included 16 patients considered hormone sensitive (HSPC) and 31 CRPC patients. AR CN in healthy control cfDNA ranged from 0.72 to 1.33 when screened using the AR-Amp-1/T877A assay (mean ± SEM: 1.08 ± 0.06) and ranged from 0.99 to 1.24 using the AR-Amp-2 assay (mean ± SEM: 1.13 ± 0.04) (Figure 3), which are statistically significantly less than those of patient samples (*p* < 0.01) and are used to determine a threshold for defining AR amplification. Conservatively, AR amplification threshold was set as 2 to ensure high specificity of higher AR CN calls. In our patient cohort (*n* = 47), nine patients showed cfDNA based evidence of increased AR CN. Importantly, AR amplification was consistently detected with both methods, ranging from 3.93 to 48.13 fold of amplification detected using the AR-Amp-1/T877A assay (mean ± SEM: 3.74 ± 1.32) and 2.91 to 35.59 using the AR-Amp-2 assay (mean ± SEM: 2.98 ± 0.92), while the remaining patients show normal AR CN similar to healthy controls 0.82–1.92 (mean ± SEM are 1.14 ± 0.03 and 1.05 ± 0.02 for two methods, respectively) (Figure S2). CN of AR amplified patients is significantly higher than those of normal AR CN patients and healthy controls (*p* < 0.0005), which further validated our threshold setting and suggests reliable detection of AR CN in PC patients. Also in this cohort, none of the HSPC patients (*n* = 16) show AR amplification and 29% (9/31) of CRPC patients have detectable AR amplification. It is noteworthy that both methods show very strong correlation (Pearson r = 0.94, *p* < 0.0001) and thereby present a critical way of internal cross-validation of cfDNA derived AR CN data (Figure 4). The AR-Amp-1/T877A assay also identified the T877A mutation in cfDNA from two patients; interestingly, though, both patients were considered to have HSPC at the time of blood draw (Figure 4).

**Figure 5:**
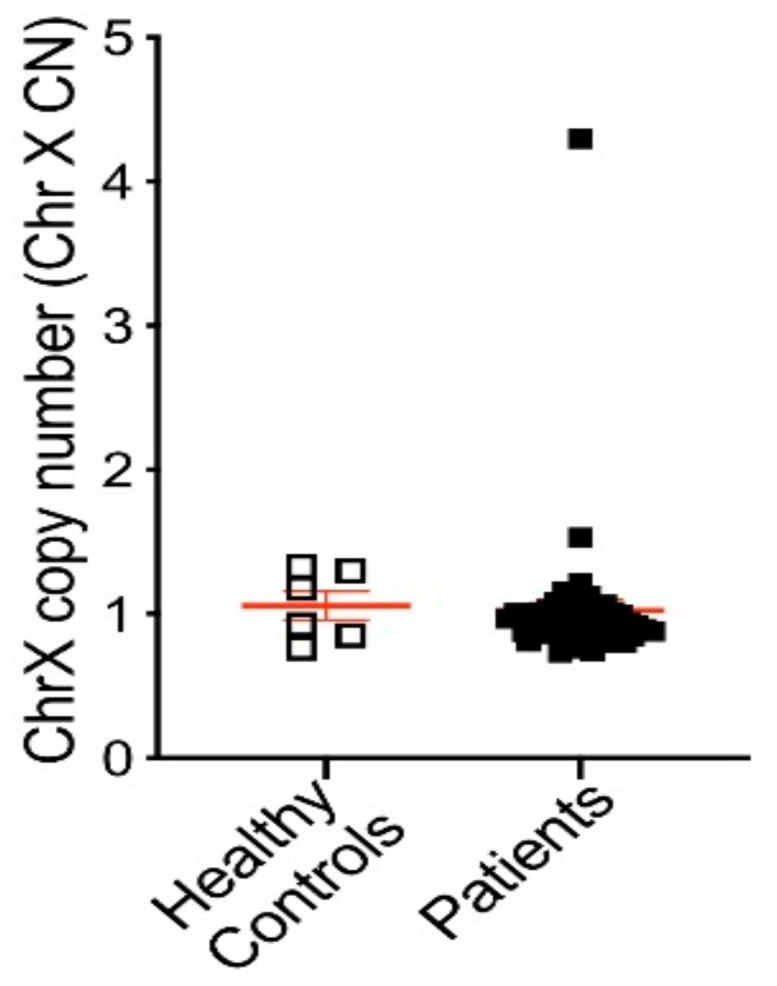
Chr X CN of 6 healthy controls and 47 PC patients as determined with AR-Amp-2 method. The threshold was set at 2. Chr X: X chromosome.

The AR-Amp-2 assay, by including an X-chromosomal (Chr X) reference gene, not only provides the information of AR CN but indicates potentially larger ChrX amplifications (MYM and AR are located on chromosome X, with a distance of ~3.6 megabases). Chr X copy number can be calculated via the ratio of two times MYM vs. TBP (Chr X CN = 2 × MYM/TBP). In one patient, we detected increased copy numbers of both MYM and AR indicating major Chr X amplification (CN of Chr X: 4.30), also indicating possibly heterogeneous Chr X amplifications considering that cfDNA is a pool DNA released by cancer and normal cells (Figure 5).

### 3.4. Association of AR^amp+^ and Treatment

We further examined whether AR CN status correlates with previous treatments in 30 CRPC patients (one patient had missing treatment information). In the patients with previous chemotherapy, 42% (5/12) were AR amplified compared to 22% (4/18) of patients with no previous chemotherapy. For patients that have received second generation ADT (enzalutamide or abiraterone) at some stage during prior therapy, 29% (4/14) were AR amplified compared to 31% (5/16) of patients that never received second generation ADT treatment. In the patients with both chemotherapy and second generation ADT, 50% (2/4) had increased AR CN compared to 27% (7/26) patients who never received chemotherapy or second generation ADT. While these data may show interesting trends, there was no signification correlation between AR CN status and any of the treatment regimens in our small cohort by Fisher’s exact test (see Table 4).

## 4. Discussion

AR amplification is rarely detected in HSPC (around 1%); however, the incidence is increased to about 30–50% in CRPC with CN gains as high as 56 copies [29]. Importantly, AR amplification is associated with shorter progression free survival [30]. AR CN measurement is therefore a proposed ADT resistance biomarker; unfortunately, at the advanced PC stage, tumor tissue biopsies are rarely available to test AR CN to inform patient management. Detection of AR CN from liquid biopsies, in particular from cfDNA, is an attractive alternative that could be easily included in patient monitoring.

Detection of point mutational biomarkers from cfDNA with ddPCR is common practice and becoming part of clinical management, for example, testing EGFR mutations in non-small cell lung cancer [26]. The overall low proportion of circulating tumor DNA (ctDNA) in a mostly wild type, normal cell derived, pool of cfDNA makes definite identification of gene amplifications extremely challenging. The variable concentration of tumor DNA in any given cfDNA sample requires the ability of cross-validation to ensure that appropriate thresholds are set to confidently call amplification versus normal CN [31]. To address this issue, we developed two independent ddPCR based methods that target three distinct regions of the AR gene together with three independent reference genes to allow sensitive and reliable identification of AR amplification. Additionally, we included detection of the AR T877A mutation in one of our assays. Although point mutations overall play a less prominent role in ADT resistant compared to AR amplification status and AR splice variant V7, AR T877A single mutation or double mutation AR W741C-T877A or AR F876L-T877A has been reported to switch hydroxyflutamide from an AR antagonist to agonist, and T877A is likely an underlying mechanism of resistance to flutamide [12]. Therefore, detection of T877A is of clinical importance.

In addition, 10–40% of PC patients have been reported to have entire chromosome X amplification and additional AR copies have been reported even in treatment naive patients due to polysomy of chromosome X [32]. Increased Chr X CN with AR amplification in primary PC tends to correlate with a higher tumor stage and Gleason score and poorer survival; however, the reported case number remains low [29,33]. Most AR CN studies were performed with fluorescent in-situ hybridization (FISH) on tissue biopsy [33,34], which apply both AR and other Chr X probes to visualize CN changes in individual cells. The AR-Amp2 assay indicates ddPCR detectability of Chr X CN changes from cfDNA; however, this needs further validation. In addition, more studies are warranted on the association of patient outcome and AR CN changes, including higher vs. lower AR CN gain measured with our methods from cfDNA.

We applied our assays to a cohort of 47 PC patients to test translatability and found AR amplification in nine patients, with sometimes very high amplification of the AR gene. The highest AR CN detected by the AR-Amp-1/T877A assay was 48. According to our corresponding AR-Amp-2 assay data, this amplification can at least in part be explained by larger scale X-chromosomal amplifications as the distant X-chromosomal reference gene MYM was also clearly increased over the TBP reference gene in the patient. AR CN gain detection with our assays was restricted to CRPC patients (29%, 9/31). These data agree with another study which examined the prognostic relevance of plasma cfDNA based detection of AR amplification in pre-chemotherapy metastatic CRPC and found evidence for AR CN gain in 27% of patients [22], while AR CN gain was reported for 40% of chemotherapy-treated CRPC patients in another study [35]. We also saw a trend, albeit not significant, for AR CN gain association with previous chemotherapy, while in a previous study testing AR CN gain with array comparative genomic hybridization (aCGH), AR CN was more common in patients progressing on enzalutamide (*n* = 19, 53%) than on abiraterone (*n* = 29, 17%) and other agents (*n* = 14, 21%) [8].

DdPCR is one of the most sensitive and economic ways to detect rare gene point mutations, amplification, and expression levels. Compared to FISH or Whole genome sequencing (WGS), ddPCR is more time- and cost-economic with superior sensitivity despite the superiority of FISH in discriminating cell-cell heterogeneity and WGS in wider coverage [36]. Given its sensitivity and relatively low costs, it is the method of choice for cfDNA based biomarker detection in a clinical setting. However, ddPCR utility in gene CN variation from cfDNA is challenging, mainly because the proportion of ctDNA in cfDNA may be low and overall variable. Additionally, certain DNA sequences may be prone to being underrepresented or overrepresented in cfDNA due to nucleosome structures and localizations. Furthermore, cancer cell genetic instability may also affect the abundance of reference genes (for example MYM), and multiple reference genes should be considered for accuracy. Here, we employed two assays with three reference genes and tested AR gene copy number at three distinct AR loci to increase reliability of AR CN detection from cfDNA. VCaP and LNCaP copy number measured with both our assays are similar to previous reports highlighting our ability to detect AR CN gain [23]. In our patient cohort, both methods discriminate high AR CN patients from normal AR CN patients and healthy blood donors and show very high correlation (*p* < 0.0005). Thus, we conclude that our assays, especially if used in combination, show high sensitivity and specificity.

The limitations of this study include testing the assays on a relatively small number of patients which were heterogeneous in the disease stage and treatment schedules. Therefore, correlation between AR CN status and disease progression still needs to be evaluated in a larger more homogeneous patient cohort over time.

## 5. Conclusions

We have established two multiplexed ddPCR based methods to detect AR CN changes from cfDNA. A simple combination of both assays produces a high security to call AR amplification from cfDNA samples that can be obtained longitudinally during PC patient management. Reliable and sensitive detection of AR amplification and T877A status may have application for patient monitoring and to define eligibility to certain clinical trials. Larger studies are warranted to define correlation of AR CN status with treatment types and disease outcomes.

## Figures and Tables

**Table 1:** Primers and probes with working concentrations in brackets.

	Primers	Probes
AR Exon 8 (AR-X8)	FP:5′-CCCTACAGATTGCGAGAGAGC-3′(500 nM) RP: 5′-GAAAGTCCACGCTCACCATGT-3′(500 nM)	MT:5′-[6FAM]ATCAGTTCGCTTTTGACCTG[BHQ1]-3′(250 nM) WT: 5′-[HEX]ATCAGTTCACTTTTGACCTG[BHQ1]-3′(250 nM)
RPP30	FP:5′-GATTTGGACCTGCGAGCG-3′(500 nM) RP: 5′-GCGGCTGTCTCCACAAGT-3′(500 nM)	5′-[HEX]TCTGACCTGAAGGCTCTG[BHQ1]-3′ (250 nM)
AR Exon 1 (AR-X1)	FP:5′-CCTATGCAAATGCCTGCCTG-3′(500 nM) RP: 5′-GCACTCTGCATTCGTTTCCC-3′(500 nM)	5′-[6FAM]AAGTCCGGTACAAAGCCAG[BHQ1]-3′(125 nM)
AR Exon 2 (AR-X2)	FP:5′-TTTCCACCCCAGAAGACCTG-3′(500 nM) RP: 5′-AAGACCTTGCAGCTTCCACA-3′(500 nM)	5′-[6FAM]CACCCAGAAGCTTCATCTC[BHQ1]-3′(250 nM)
MYM	FP:5′-ACAGGGAACAGAACAAGCTGGTCTT-3′(1000 nM) RP:5′-GCAAGACCCTGTGTAAGAACTTTGA-3′(1000 nM)	5′-[HEX]CATTACGATCCACATGTGATAG[BHQ]-3′(250 nM)
TBP	FP:5′-ACAGAAGTTGGGTTTTCCAGC-3′(500 nM) RP: 5′-TCACATCACAGCTCCCCAC-3′(500 nM)	5′-[HEX]TCTTGGACTTCAAGATTCAG[BHQ1]-3′(500 nM)

Reference genes: *RPP30* (ribonuclease P/MRP subunit p30, chromosome 10: 2 alleles per normal cell), TBP (TATA-binding protein, chromosome 6: 2 alleles per normal cell), MYM (zinc finger MYM-type protein 3, X chromosome; 1 allele per normal male cell).

**Table 2:** AR-Amp-1/T877A assay using gDNA from PBMCs and cell lines as well as mixed LNCaP and PBMC gDNA (concentration unit for each gene: copy/µL; M: male; F: female).

Cell Sample (gDNA)	T877A	WT	RPP30	AR:RPP30	AR CN
PBMC-M	0	45.4	90	0.51:1	1
PBMC-F	0	81	77	1.05:1	2
MFM-223	0	198	51.5	3.84:1	8
VCaP	0	721	52.5	13.73:1	28
LNCaP	114	0	228	0.50:1	1
LNCaP + PBMC-M (2:1)	30.1	16.2	89.3	0.52:1	1.0
LNCaP + MFM-223 (2:1)	29	60.7	68.7	1.31:1	2.6
LNCaP + VCaP (2:1)	20.4	191	61.7	3.43:1	6.9

**Table 3:** AR-Amp-2 assay of gDNA from PBMCs and cell lines (concentration unit for each gene: copy/µL; M: male; F: female; AR CN as calculated in the Materials and Methods part, MYM* see Droplet Digital PCR part).

**Cell Sample (gDNA)**	**AR-X1**	**AR-X2**	**TBP**	MYM*	**AR CN**
PBMC-M	45.9	42	87	84.6	1
PBMC-F	73	72.6	73.1	75	2
MFM-223	173	176	46.3	51.8	7
VCaP	696	709	54	69	23
LNCaP	97	100	189	194	1

**Table 4:** Association of plasma AR^amp^^+^ status with treatments.

	Treatment Type						
	Chem+	Chem−	Enz/Abi+	Enz/Abi−	Chem and Enz/Abi+	Others	Total
High AR CN	5	4	4	5	2	7	9
Normal AR CN	7	14	10	11	2	19	21
Total	12	18	14	16	4	26	30

Note, all patients (*n* = 30) had previous first line ADT treatment. Chem: chemotherapy; Enz/Abi+: enzalutamide or abiraterone. Fisher’s exact test, not significant in all groups (Chem+ vs. Chem-, *p* = 0.42; Enz/Abi+ vs. Enz/Abi−, *p* = 0.99; Chem and Enz/Abi+ vs. others, *p* = 0.56).

## Data Availability

Data available on request due to restrictions e.g., privacy or ethical.

## Supplementary Materials

The following are available online at https://www.mdpi.com/article/10.3390/jcm11010257/s1, Table S1: Patient information and raw ddPCR data, Figure S1: Optimization of AR-Amp-2 with various concentrations of primers and probes for TBP, MYM (A,C) and AR-X1, AR-X2(B). Figure S2: Dot plots showing the comparison of amplification positive with negative patients and healthy controls with two methods. *p* < 0.0005.
