# Supplementary figures and images for: Biomarkers of Castrate Resistance in Prostate Cancer: Androgen Receptor Amplification and T877A Mutation Detection by Multiplex Droplet Digital PCR
Source: J Clin Med. 2022 Jan 4;11(1):257. doi: 10.3390/jcm11010257 (PMC8745706; doi:10.3390/jcm11010257)

Figure S1.

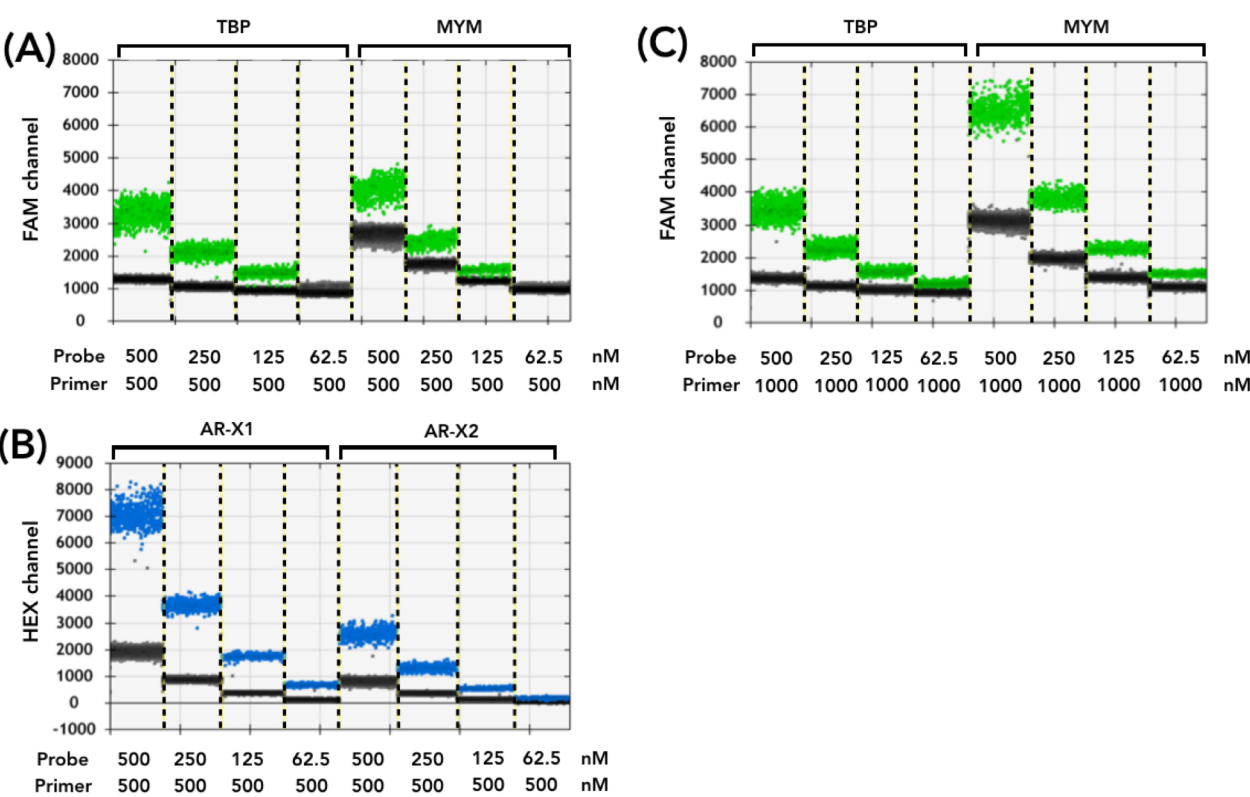

Figure S2.

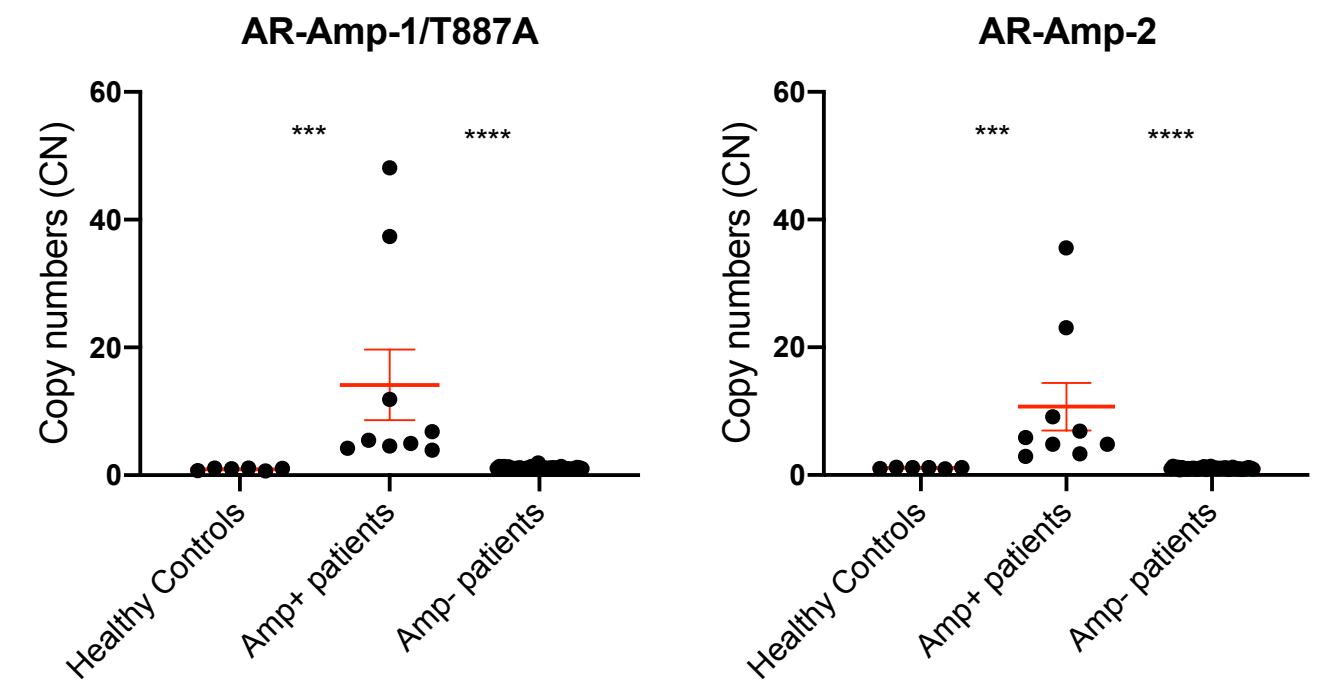

Supplement: Supplementary file 1 [file jcm-11-00257-s001.zip › Supplementary figures.pdf]
